# Effect of Temperature and Humidity Coupling on the Ageing Failure of Carbon Fiber Composite/Titanium Bonded Joints

**DOI:** 10.3390/polym16070952

**Published:** 2024-03-30

**Authors:** Han Peng, Tai Zhou, Linjian Shangguan, Ruixue Cheng

**Affiliations:** 1 School of Mechanical Engineering, North China University of Water Resources and Electric Power, Zhengzhou 450045, China; 18637720887@163.com; 2 School of Computer Engineering and Digital Technology, Teesside University, Middlesbrough TS1 3BA, UK; r.cheng@tees.ac.uk

**Keywords:** adhesive-bonded joints, moisture–heat aging, failure intensity, reliability analysis

## Abstract

Temperature and humidity coupling has a more significant effect on the failure properties of bonded joints than a single factor, and there is not enough research on this. In this paper, joints bonded with strong toughness structural adhesives are selected for the experimental analysis of joints aged for 240 h, 480 h, and 720 h at temperatures of 40 °C and 60 °C and a humidity of 95% and 100%. The sequential double Fick’s model was used to fit the water absorption of the joints, and the comparison yielded that the water absorption of the adhesive was in accordance with Fick’s law. The quasi-static tensile tests revealed that the reduction in mechanical properties of the joints was positively correlated with the moisture content in the environment, while the competing mechanisms of post-temperature curing and hydroplasticization resulted in a slight increase in the failure strength and energy uptake of the aged joints, which is in agreement with the experimental results of the Fourier infrared spectroscopy. A combination of macroscopic failure sections and scanning electron microscope (SEM) images yielded that the failure mode of the joints changed from cohesive failure to interfacial failure with increasing ageing time. In addition, reliability analyses for the fatigue testing of joints are expected to provide guidance for the life design of bonding technology in the vehicle service temperature range.

## 1. Introduction

In the face of severe global energy and climate problems, the development of lightweight structures that reduce energy consumption and carbon monoxide emission pollution is slowly gaining worldwide attention [1,2]. The lightweighting of automobiles helps them reduce carbon emissions, improve dynamics, etc. [3]. Therefore, exploring automotive lightweight materials is a top priority to achieve a more efficient structural design of future vehicles [4,5,6]. Lightweight technology is primarily divided into three aspects: structural optimization, the application of lightweight materials, and advanced manufacturing processes [7]. Among them, the application of lightweight materials is the most convenient and potential method for automobile lightweighting. The use of FRP-type composites in lightweight materials is becoming a trend, and the advantages of using FRPs (fiber-reinforced polymers) over steel are 20–40% weight reduction; 40–60% reduction in tooling costs; flexible styling compared to deep-drawn panels; resistance to corrosion, scratches, and dents; reduction in assembly costs and integration time; reduction in noise and vibration, and higher damping; innovation in materials and processes adding value and saving money; and providing a safer automotive structure absorbing a higher amount of specific energy in the event of a collision (SEA, a key metric in the design of vehicle crashworthiness) [8]. Carbon fiber-reinforced polymer (CFRP) composites are used widely in the automotive industry for advantages such as high specific strength, high specific modulus, good corrosion resistance, fatigue resistance, etc. [9]. However, the application of CFRP poses a challenge to the joining technology between different materials.

Adhesive bonding is one of the most commonly used joining techniques for composite materials [10,11]. Traditional joining techniques such as welding, riveting, and bolting not only destroy the carbon fiber integrity of CFRP, but the presence of holes also causes stress concentrations [12]. Bonding technology is a method of bonding materials together through intermolecular forces or chemical reactions. Its advantages include the following: it can effectively make up for connections that are difficult to achieve with traditional processing techniques such as welding, it does not damage the surface of the materials to be joined, and it does not produce inferior phenomena such as thermal deformation; it also improves the service life of the materials, durability, and so on. It is also suitable for the connection of FRP-type materials. Bonding technology is widely used for joining materials in vehicles. Bonded structures are subjected to a variety of factors such as temperature, humidity, and UV exposure in real-world environmental applications [13], and the different factors are often superimposed on each other. Temperature and humidity coupling (hydrothermal environments) are usually more influential than individual factors [14]. Previous studies have shown that the mechanical and adhesive properties of bonded joints are not significantly affected by low-temperature ageing, and that the thermal stresses caused by the different coefficients of thermal expansion of the adhesive and the bonded substrate can lead to slight fluctuations in the failure strength of the bonded joints [15,16,17]. As the aging temperature increases, the mechanical and adhesive properties of bonded joints decrease, which is due to the thermal decomposition and oxidation reaction of composites and adhesives at high temperatures [18,19,20]. Therefore, research related to the hygrothermal environments of bonded structures is necessary.

Bonded joints are often subjected to harsh environments during their service life, with temperature changes being the most common and one of the most important parameters, as the bonding agent’s tensile strength, Young’s modulus, and strain tend to change significantly with temperature changes [21]. In addition, the surface treatment of the bonding substrate, the addition of nano/particle fillers to the bonding agent, and the sealing treatment of the bonding area can be effective in improving bonding performance. Yao et al. [22] analyzed the temperature effects on BFRP-steel single lap joints, and a significant effect of high temperature on mechanical properties was observed, as they found that the average bond strength increased in the −25 to 50 °C range but decreased significantly in the 50 to 100 °C range, and shear stiffness increased in the −25 to 25 °C range but decreased in the 25 to 100 °C range. In a study by Bai et al. [23], CFRP–steel bonded joints were found to lose about 80% or so of their stiffness and strength at temperatures near or above their glass transition temperature. To evaluate the durability of low-pressure plasma surface treatment on bonded joints under accelerated aging at high temperatures and high humidity, Pizzorni et al. [24] conducted shear strength tests and wedge cut tests on accelerated aged joints, and their results showed that the low-pressure plasma surface treatment method can improve the quality of bonded joints in the short term, and increase their durability even under severe aging conditions. Akman et al. [25] performed a comparison of photochemical and photothermal laser ablation on CFRP/CFRP bonded joints in terms of shear strength, and it was found that both laser surface treatments had a definite enhancement of the bond strength. The rapid UV irradiation [26] of composite substrates also significantly improved the integrity of bonded joints. For the surface treatment of metal substrates in addition to sandblasting or sanding to change the surface roughness, Rudawska et al. [27] compared the effects of chromate, phosphate, and smooth coatings on the performance of bonded joints of hot-dipped galvanized steel sheets, expressed the change in bonding surfaces in terms of surface roughness, and found that smooth coatings had the worst performance of bonded joints. Moreover, the addition of carbon nanotubes to the adhesive improved the mechanical properties of bonded joints to some extent and enhanced the thermal cycling properties of joints. The researchers not only discussed the effect of the concentration of carbon nanotubes on the bonded joints but also toughened the bonded joints by functionalizing the carbon nanotubes.

In the service process, the automobile bonding structure is subjected to temperature changes in the general range of −40~80 °C [28], which requires the bonding structure to have good temperature resistance, to ensure that its residual strength is not less than the safe permissible value both in the short and in the long term. Therefore, it is essential to study the effect of moisture–heat aging on the mechanical properties of carbon fiber composites and titanium alloy joints to better understand the performance of different bonding materials under different stresses and environments so as to guide the selection and optimization of materials and improve the performance and reliability of bonded joints. At present, there is still a lack of research on the failure strength and failure mechanism of adhesive joints under environmental aging at home and abroad, and most of the existing studies are aimed at the mechanical properties of single lap joints composed of CFRP, metallic aluminum, and high-strength steel in hot and humid environments, while there are relatively fewer studies on mechanical properties of single-lap joints composed of CFRP and titanium alloys in hot and humid environments. Based on the above, in this paper, CFRP and TC4 titanium alloy, which are commonly used materials in the automotive field, are bonded to form a single-lap joint, using the automotive structural epoxy resin adhesive Araldite^®^ 2015 (EXP2015). According to the automotive conditions, 40 °C and 60 °C ambient temperature, 95% RH (relative humidity), and 100% RH (relative humidity) are selected as the environmental conditions of hot and humid coupling, and 240 h, 480 h, and 720 h aging tests are carried out for the bonded joints under hot and humid environments. A quasi-static tensile test is used to analyze different aging times under joint failure strength, maximum failure load, and reliability. A “dumbbell-type” adhesive was fabricated and tested for water absorption, and the relationship between failure strength and water absorption concentration of the adhesive was obtained. To investigate the failure strength and failure mechanism of adhesive joints in hot and humid environments, and to explain the aging behaviour of adhesive joints in hot and humid environments by using macroscopic and microscopic tests.

## 2. Experimental Step

### 2.1. Preparation of Test Pieces

#### 2.1.1. Material Selection

A CFRP sheet was made of T700 fiber and BA9916 epoxy resin, using prepreg processing technology. The layup mode was (0/90/0/90), with a total of 10 layers, at a thickness of 2 mm, and its mechanical property parameters are shown in Table 1. The epoxy Araldite^®^2015(EXP2015) was selected as the adhesive (Huntsman Advanced Materials Ltd., The Woodlands, TX, USA), and the ratio of epoxy resin and curing agent was 1:1. Araldite^®^ 2015 is a tough adhesive with very high lap shear, stripping strength, and resistance to dynamic loading; its concrete parameters are shown in Table 2. Ti-6Al-4V (TC4) titanium alloy was selected as the metal base material, which has advantages of plasticity, high thermal strength, low-temperature working performance, strong corrosion resistance, etc., and is widely used in the automotive industry. Its alloy composition is 5.5–6.75 wt% Al, 3.5–4.5 wt% V, 0.3 wt% Fe, 0.2 wt% O, 0.08 wt% C, 0.05 wt% N, 0.015 wt% H, and the remaining element is Ti.

#### 2.1.2. Single-Lap Joint (SLJ)

**Figure 1:**
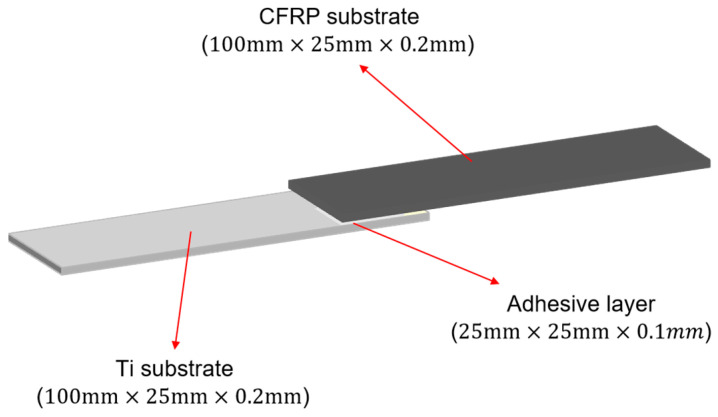
Single-lap joint geometry (mm).

The standard reference for the production of bonded joints is ISO 4587:2003 [30]. Firstly, the CFRP substrate with a size of 100 mm × 25 mm × 2 mm was matched with the Ti substrate; after that, the surface of the Ti substrate was sandblasted (#80 aluminum oxide sandblasting time of 5–10 s; the purpose is to increase the roughness of the surface, and improve the contact area and mechanical interlocking between the adhesive and adhered object), and then the surface was sandblasted for 5–10 s. As sandblasting would cause irreparable damage to the fibers and resins of the CFRP substrate, CFRP was not sandblasted; to prevent the effects of grease and dust on the surface of the substrate on the adhesive adsorption, this was followed by acetone cleaning treatment of the surface of the CFRP and Ti substrates. After 10–15 min of static at room temperature, the CFRP was subjected to the plasma surface treatment process (parameter selection: a power of 550 W, and a speed of 1.5 mm/s); the two-component glue gun was used for glue application, and a jig was used to complete the bonding joint. After the bonding was completed, the specimens were taken down after curing for 24 h at room temperature (25 ± 3 °C), excess glue tumors of the specimen were removed, and the impact of the glue tumors on the performance of the bonded joints was eliminated to complete the fabrication of the joints. The specific specimen size is shown in Figure 1.

#### 2.1.3. Adhesive

**Figure 2:**
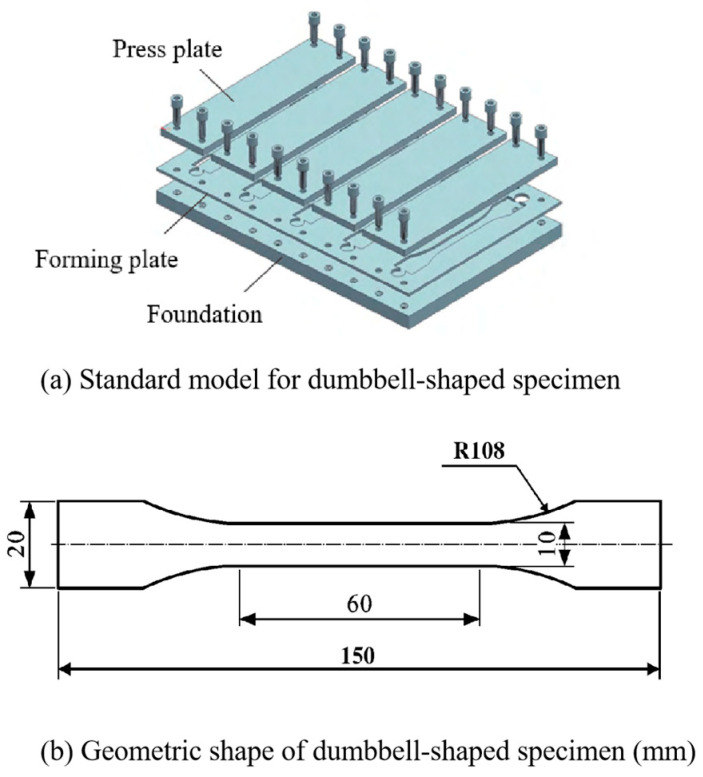
Dumbbell-shaped specimen.

Considering that the adhesive inevitably absorbs water and swells under high humidity, a water absorption test of the adhesive body under four environments was conducted. The adhesive body was fabricated according to NF ISO 527-2-2012 [31] as a dumbbell-shaped specimen with 2 mm thickness, and the exact dimensions and shapes are shown in Figure 2. The fabrication process of the dumbbell-shaped specimen is as follows:

(1) Open the humid–heat alternating test chamber in advance and adjust the temperature to the temperature at which the specimen is cured;
(2) Load EXP2015 into the glue gun and punch it into the PTFE mold that has been wiped with acetone in advance (the purpose of treating the mold with acetone is to remove oil and dust from the surface of the mold to prevent air bubbles and sticking phenomena from occurring in the specimen made at a later stage due to the influence of the dust particles), and then cover it with a cover plate;
(3) EXP2015 which is in the flow state in the mould is put into the hot and humid test chamber for curing together with the mould. wait until the end of curing, take out the cured dumbbell specimen, and carry out the aging experiments in different environments of 240 h, 480 h, and 720 h.

### 2.2. Test Methods

#### 2.2.1. Damp Heat Aging Test

In order to investigate the effect of the humidity-heat coupling environment on the failure strength and failure mechanism of EPX2015 joints, a damp heat aging test was performed in accordance with GB/T 41767 [32]. This experiment sets three different aging time limits of 240 h, 480 h, and 720 h in each aging environment; sets the unaged group as the control group, with a total of 13 experimental groups; and selects three specimens of better quality and uniform standard for each group. Before the aging test, the specimens were grouped into a high-temperature box at 80 °C for high-temperature curing for 2 h, and then placed at cooling room temperature into the high-low temperature hot and humid test box (Weiss Equipment Experiment Company, Frankenmuth, MI, USA, WS-1000). Set 40 °C/95% RH, 40 °C/100% RH, 60 °C/95% RH and 60 °C/100% RH four kinds of hot and humid environment to reach the corresponding aging time will be taken out of the specimen for the next step of experimental testing. 

#### 2.2.2. Water Absorption Test

To compare more intuitively the effects of water on EXP2015 adhesive performance in an aging environment, this experiment was conducted to test the water absorption of EXP2015 dumbbell-type standard parts. Using weight measurement to obtain moisture absorption $M_{t}$, the use of high-precision analytical scales with an accuracy of 0.1 mg every 24 h on the specimens in the high and low temperature humidity and heat alternating experimental chamber measured once, before measuring the weight of the absorbent paper will be used to absorbent paper on the surface of the standard parts of the water wiped clean. The entire test process needs to be as short as possible. The purpose of this test is to avoid the other interferences affecting the accuracy of the experiment. The water absorption formula is as follows:

(1) $$M_{t}=\frac{W_{t}-W_{0}}{W_{0}}\times 100\%$$

where *M_t_* denotes the water absorption at time *t*, *W_t_* denotes the mass at time *t*, and *W*_0_ denotes the initial mass.

#### 2.2.3. Quasi-Static Tensile Testing

**Figure 3:**
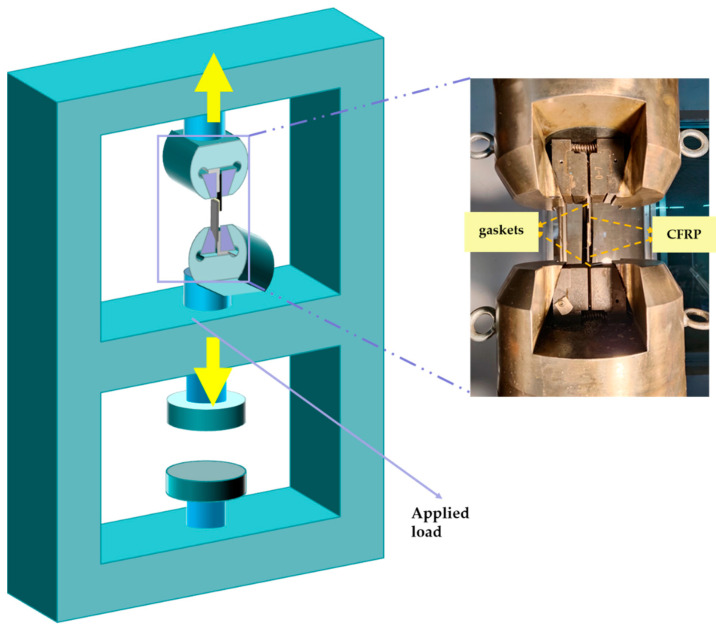
Schematic diagram of Xin Guang universal tensile testing machine.

To test EPX2015 joints’ mechanical properties, specimens that reached a specified aging time were removed for static tensile testing. Figure 3 shows the schematic diagram of the quasi-static tensile process of CFRP-Ti joints on the Xin Guang universal test machine (China Jinan Xin Guang Testing Machine Manufacturing Co., Ltd., Jinan, China). To prevent bending stresses during the stretching of joints, referring to ASTM D1002 [33], shims with 2 mm thickness were inserted at both ends of the specimens, and joints were stretched to destruction at the constant tensile rate for 2 mm/min, and load–displacement curves and maximum failure strengths during stretching were recorded by the computer.

#### 2.2.4. Fourier-Transform Infrared Ray Spectroscopy (FTIR) Tests

The structure of the EXP2015 adhesive was analysed by FTIR [34] and the sample surface was spectroscopically analysed using a VERTEX70V Fourier Transform Infrared Spectrometer, with samples extracted from the bonding area. Spectra were obtained using the attenuated total reflection (ATR) method for 128 scans. Before analysis, the spectrometer platform was cleaned with dry air. The spectral range was 500–4000 cm^−1^ with a resolution of 4 cm^−1^ for 128 scans.

#### 2.2.5. Reliability Analysis

The ability of a bonded joint to fulfill a specified function throughout its life cycle in practical engineering applications can be obtained using statistical studies, and many statistical models can be used for reliability studies, focusing on normal, Poisson, exponential, and Weibull distributions [35]. The Weibull distribution is very common in industrial applications such as aerospace or automotive because of its good compatibility. In this study, the reliability percentage of stresses was obtained by creating a simplified Weibull distribution, which allows the calculation of failure risk to joints, despite the unavailability of the cause of the joint failure. The superior performance of the joint was further evaluated by comparing the failure risk of two joints.

## 3. Experimental Results

### 3.1. Moisture Absorption

To study the response of moisture absorption and diffusion of adhesives during aging, in general, the moisture absorption process of adhesives may be described using Fick’s law [36]. One of the simplest diffusion models is Fick’s law, assuming that the polymer chains do not react with moisture and can be observed in epoxy resins well above Tg [37]. Due to the influence of specimen geometry, size and material properties, the Fick diffusion behaviour of the adhesive specimen is inevitable in the process of absorbing water, so the simple Fick’s law has some limitations in describing the water-absorbing behaviour of the adhesive specimen. In this study, the sequential double Fick’s model [38] (SDF), applicable to toughened epoxy adhesives, was used to fit the experimental data, and the derivation of its equations is as follows.

In the simple Fick’s law model, the water diffusion analytical equation can be expressed as:

(2) $$M_{t}=M_{\infty }\times \left[1-\frac{8}{\pi^{2}}\sum_{n=0}^{\infty }\frac{1}{{\left(2n+1\right)}^{2}}\exp(-\frac{{\left(2n+1\right)}^{2}\pi^{2}D}{h^{2}}t)\right]$$

where *M_t_* is the amount of water absorbed at moment *t*, *M_∞_* is the moisture content at saturation, *h* is the thickness of the specimen, *D* is the diffusion coefficient, and *t* is the moisture absorption time.

The SDF model is a superposition of two single Fick’s laws, which assumes that Fick’s law acts in two successive stages of water absorption; that the two successive stages have different diffusion properties, i.e., that each stage has its diffusion coefficient and saturated water concentration; and that, in addition, the model assumes the existence of a pseudo-equilibrium between the first diffusion mechanism and the second diffusion mechanism. In this model, the analytical equation for water diffusion can be expressed as:

(3) $$M_{t}=M_{1\infty }\times \left[1-\frac{8}{\pi^{2}}\sum_{n=0}^{\infty }\frac{1}{{\left(2n+1\right)}^{2}}\exp\left(-\frac{{\left(2n+1\right)}^{2}\pi^{2}D_{1}}{h^{2}}t\right)\right]+\phi \left(t-t_{d}\right)\times M_{2\infty }\;\times \left[1-\frac{8}{\pi^{2}}\sum_{n=0}^{\infty }\frac{1}{{\left(2n+1\right)}^{2}}\exp\left(-\frac{{\left(2n+1\right)}^{2}\pi^{2}D_{2}}{h^{2}}\left(t-t_{d}\right)\right)\right]$$

where $M_{1\infty }$ and $M_{2\infty }$ are the saturated water uptake in the first and second stages, $D_{1}$ and $D_{2}$ are the diffusion coefficients in the first and second stages, respectively, and $M_{1\infty }$ + $M_{2\infty }$ = $M_{\infty }$. $t_{d}$ is the moment when the first diffusion mechanism is transformed to the second diffusion mechanism, and $\phi (t-t_{d})$ is the step function, defined as:

(4) $$\phi (t-t_{d})=\left\{\begin{array}{c} 0,\;t<t_{d}\\ 1,\;t\geq t_{d} \end{array}\right.$$

The SDF model parameters of the EXP2015 adhesive in different aging environments were obtained from the experimental data by fitting Equations (2) and (3) using the least squares method in MATLAB(R2022a) (Table 3).

**Figure 4:**
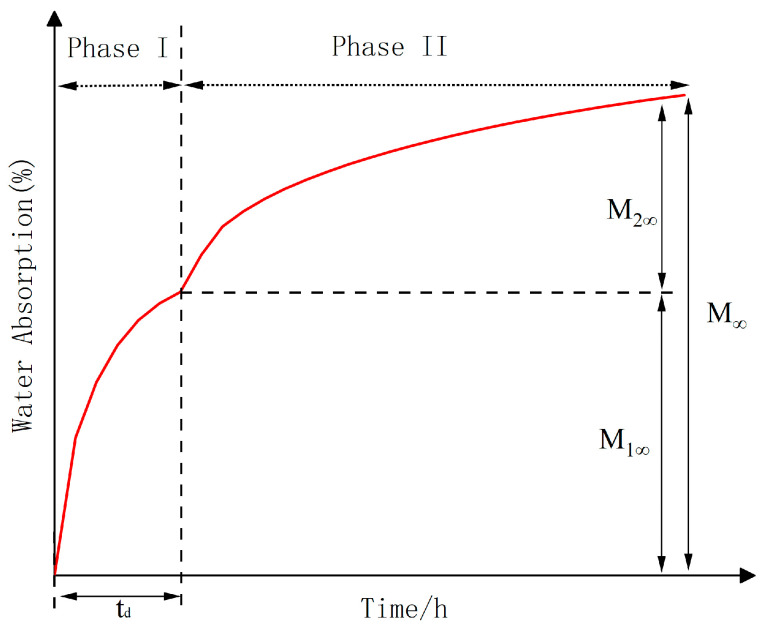
Schematic diagram of the sequential block model. (The moisture absorption curve was divided into two phases by making dotted lines based on *t_d_*.)

Several studies have elaborated on the formation and presence of water molecules in epoxy resins in two states. One by diffusion of water into the epoxy resin and in the free volume (pores, cavities mainly) of the material [39].The other is the strong coupling of water molecules with certain hydrophilic groups (e.g., hydrogen bonds) in the epoxy resin [40]. Figure 4 shows a schematic diagram of the water absorption process in the SDF model. It has been pointed out [41] that the diffusion mechanism in the first stage was affected by hydrogen bonding. The diffusion corresponding to the strong interaction of the water molecules with the hydrophilic functional group in epoxy resin belongs to the chemical mechanism. The second stage of the diffusion mechanism is related to the aggregation of water molecules, which filled the free volume in the epoxy resin. Water molecules in clusters do not have a strong connection to the polymer chain, which is essentially free, so the second diffusion mechanism in the SDF model belongs to the physical mechanism. Under the influence of hot and humid environments, studies have shown that both physical and chemical mechanisms may occur at the same time.

**Figure 5:**
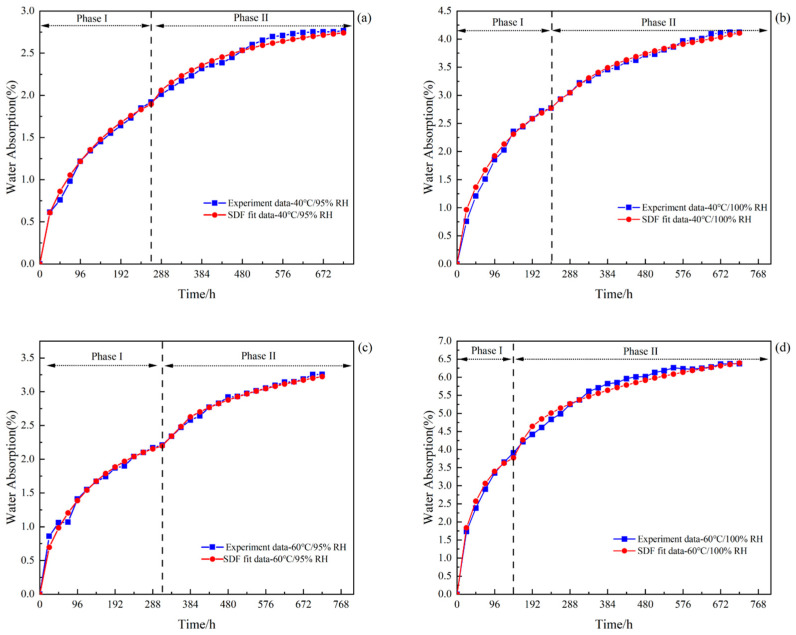
Experimental and fitted moisture absorption curves for adhesive specimens: (**a**) 40 °C/95% RH; (**b**) 40 °C/100% RH; (**c**) 60 °C/95% RH; and (**d**) 60 °C/100% RH.

Figure 5 shows the experimental and model fitting results of water absorption over time for the EXP2015 adhesive in the aging environments of 40 °C/95% RH, 40 °C/100% RH, 60 °C/95% RH, and 60 °C/100% RH, and according to the images, it can be seen that the data fitted to the SDF model almost completely overlap with the experimental data. The moisture absorption process of adhesives in all four environments could be divided into two stages. In the first stage of the water molecules at a faster rate with the epoxy resin combination, the water absorption rate quickly reached the first stage of the saturation value of *M*_1∞_, where *M*_1∞_ in order of magnitude 3.91% (60 °C/100% RH), 2.77% (40 °C/100% RH), 2.21% (60 °C/95% RH), 1.92% (40 °C/95% RH) The water absorbency rate gradually decreased during the first stage of the process, followed by a short plateau period; with the aging time increasing, the specimen showed a significant secondary increase in water absorbency until saturation. The water absorption rate of the second stage was observed to be significantly smaller than that of the first stage, which was confirmed by the data as $D_{2}$ is significantly smaller than $D_{1}$. The water content of specimens remained relatively stable after water absorbtion saturation, which can be seen from the four experimental data curves. The saturated water absorption rate of the adhesive was the highest under the condition of 60 °C/100% RH (6.77%), and the saturated water absorption rate under the condition of 40 °C/95% RH was the lowest (2.76%). The remaining two groups are 40 °C/100% RH (4.12%) and 60 °C/95% RH (3.25%), respectively. It is worth noting that in Figure 5, at the beginning of the first stage of the diffusion model, with a large deviation from the experimental curves, there is a transition time *t_d_*.

In the first stage, the water content of the adhesive rises rapidly, which mainly results from the fact that the water content of the environment is much higher than that in the dried adhesive specimen, and the potential energy brought about by the difference in the water concentration drives the water molecules to combine with the epoxy resin in the adhesive at a faster rate. According to Table 3, it can be seen that *M*_1∞_ increases significantly with RH at the same temperature, while the same positive relationship exists between temperature and the diffusion coefficient $D_{1}$, which suggests that RH affects saturated water absorption *M*_1∞_ and temperature affects the diffusion rate $D_{1}$ in the first stage [42]. The second stage of hygrothermal attack, *M*_2∞_, is affected by a combination of RH and temperature, which can be explained through water molecule aggregation. Water molecules aggregate to form water clusters, and the osmotic pressure and thermal expansion within the water clusters can increase the potential aggregation site, thus expanding the polymer network; the free-volume water clusters and the *M*_2∞_ will also increase, both of which are dependent on RH and temperature [43]. Noting that the diffusion mechanisms of the first stage were shown by a chemical interaction between water molecules and hydrogen bonds, the time required to complete the first stage (*t_d_*) depends on the rate of the first stage ($D_{1}$). However, the transition time (*t_d_*) at 60 °C/95% RH is longer than expected, probably because in some cases, the epoxy resin absorbs water in a pseudo-equilibrium state [44] (*M*_1∞_ occurs after *t_d_*).

### 3.2. Analysis of Mechanical Properties of Joints

#### 3.2.1. Failure Load Effects

**Figure 6:**
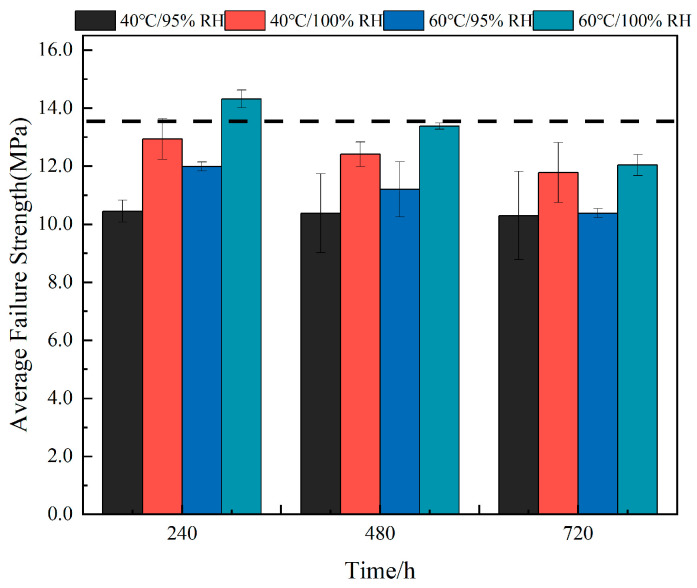
Change in mean failure strength of joints (the black dashed line represents the mean failure strength of unaged joints).

Through tensile experiments on specimens, the influence of high-temperature and high-humidity aging environments on the average failure strength of SLJ was investigated. Figure 6 shows the average failure strength of SLJ after different aging lengths under each condition, and the black dashed line represents the average failure strength of unaged joints, with a size of 13.55MPa. Compared with unaged joints, the average failure strength of joints subjected to humid and hot environments under the three aging conditions of 40 °C/95% RH, 40 °C/100% RH, and 60 °C/95% RH showed different degrees of monotonic decrease. The joints decreased most significantly at 40 °C/95% RH, and the average failure strength decreased to 22.8% (10.45 MPa), 23.4% (10.38 MPa), and 24.3% (10.30 MPa), respectively, after different time lengths of aging; at 60 °C/95% RH, the average failure strength of the joints decreased by 11.56% (11.99 MPa), 17.38% (11.20 MPa), and 23.4% (10.38 MPa); among the three aging conditions, the joints under 40 °C/100% RH aging had the least loss of failure strength, and with the increase in aging time, the average failure strength only decreased by 4.5%(12.94 MPa), 8.42%(12.41 MPa), and 13.11% (11.78 MPa). Combined with water absorption results, it can be observed that the adhesive absorbed the most water under the above three conditions at 40 °C/100% RH, and the authors concluded that more water molecules entered into the adhesive, resulting in the release of residual stress in the bonding region of the joints [43], so the joints aged at 40 °C/100% RH had the least loss of mean failure strength. Surprisingly, compared to the average failure strength of unaged joints, the average failure strength of joints at the beginning of aging at 60 °C/100% RH increased by 5.69% (14.32 MPa), but with the increase in aging time, the average failure strength of joints decreased below the black dotted line. There are two reasons for the increased failure strength of joints in the early stage of aging at 60 °C/100% RH: (Ⅰ) the highest water absorption in the joints at 60 °C/100% RH, and the more complete release of residual stresses; (Ⅱ) the exposure temperature is maintained at 60 °C, and the epoxy polymer chains or their cross-linking occur in a secondary bonding, which leads to the generation of a post-curing reaction [44], increasing the average failure strength of joints. Considering the temperature effect at the same aging time, under the same humidity, the average failure strength of joints in a 60 °C environment was higher than that of the joints under 40 °C; in terms of the effect of humidity, the average failure strength of joints under conditions of 100% RH at the same temperature is higher than that of the joints under 95% RH.

#### 3.2.2. Energy Absorption Effects

**Figure 7:**
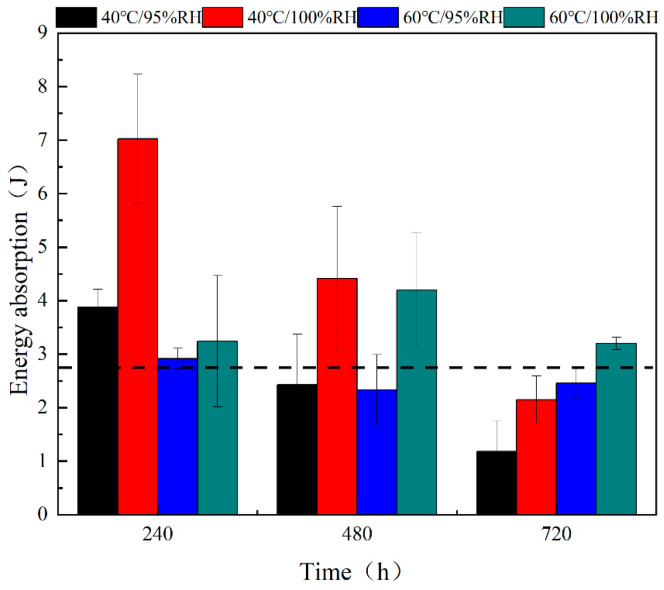
Changes in energy absorption of joints (the black dashed line represents energy absorption values for unaged joints).

A quasi-static stretch test was used to record the load–displacement curves of different joints, and calculate the energy absorption value of the joints based on the area under load–displacement curves (see Figure 7, where the black dashed line indicates the energy absorption value of unaged joints, the magnitude of which is 2.7693 J). When the aging time is 240 h, the energy absorption values of joints under the four conditions were greater than those of the unaged joints, and the order of the absorbed energy is 7.0261J (40 °C/100% RH), 3.8785J (40 °C/95% RH), 3.2401J (60 °C/100% RH), and 2.9136J (60 °C/95% RH). At 40 °C, the energy absorption values of joints monotonically decreased with increasing aging times. The energy absorbed by the joints at 100% RH during the process was always greater than the energy absorbed by the joints at 95% RH. In addition, according to the images, when the aging time exceeds 480 h, the energy absorption value of joints under 40 °C/95% RH starts to appear lower than the black dashed line; in the case of the joints under 40 °C/100% RH, the energy absorption value appears lower than the black dashed line when the aging time reaches 720 h. Under a 60 °C environment, the energy absorption value of the joints under 95% RH and 100% RH with the increase of the aging time is divided into two different stages. The energy absorption values of joints at 95% RH decrease and then increase during the aging process; however, the energy absorption values of the joints at 100% RH appear to increase and then decrease, and always remain above the black dashed line. The same is true for the 40 °C environment, where the energy absorption values at 60 °C increase with increasing RH. Before aging for 480 h, the joints at 40 °C absorbed more energy compared to 60 °C at the same humidity. In contrast, after 480 h of aging, the energy of the joints at 60 °C is higher than that at 40 °C at the same humidity.

From 0 h to 240 h, the failure strength of joints decreases with time for the three conditions of 40 °C/95% RH, 40 °C/100% RH, and 60 °C/95% RH, but the energy absorption value increases. This illustrates that while the joint stiffness decreases, the failure displacement increases, which is a typical effect of water plasticization [38]. It is interesting to note that the aging time from 0 h to 240 h is the time when the first diffusion mechanism plays a major role in the SDF hygroscopic model. When the aging time is 480 h, moisture absorption comes under the second diffusion mechanism in the SDF model, when the osmotic pressure and thermal expansion in the water clusters make the polymer network expand, weakening the stress between polymer molecules [36], leading to a further decrease in the failure strength and energy absorption of the adhesive, and a weakening of the resistance to deformation. Until 720 h, there is a small increase in the energy absorption value of joints at 60 °C/95% RH which is attributed to the softening of the resin matrix and adhesive under prolonged high-temperature aging, resulting in an increase in viscoelasticity. The trends of energy absorption and failure strength of joints at 60 °C/100% RH can be attributed to the results of competition between the two mechanisms of the post-temperature curing effect and water plasticization effect. An increase in both failure strength and energy absorption of joints is observed before aging for 240 h, and the post-temperature curing effect is more pronounced than the water plasticization effect in this aging process. In the aging time from 240 h to 480 h, the failure strength appears to decline and energy absorption appears to rise, in which the water plasticization effect was more obvious than the post-temperature curing effect. When the aging time reaches 720 h, the energy absorption value of the joint is still higher than the black dashed line, and the adhesive absorbs more water in this environment, indicating that the water-plasticizing effect continues to be significant until the end of aging.

### 3.3. Fourier Infrared Spectral Analysis

A spectral analysis of the sample surface was conducted using a Fourier-transform infrared spectrometer VERTEX 70 (Bruker, Germany). Combined with Table 4, it can be seen that the vibrational peaks of EXP2015 at 3320 cm^−1^ are –OH or –NH telescopic vibrations, and the vibrational telescopic peaks of alkyl groups (–CH_3_, –CH_2_) appeared at the interval of 3100~2800 cm^−1^. In epoxy resins, the carbonyl group (C=O) is generally present in aldehydes and amides and appears in the spectrum at the 1604 cm^−1^ vibrational peak, while 1581 cm^−1^ and 1502 cm^−1^ are the quadrant stretches of benzene rings, which are generally often stabilized in the reaction, and therefore the corresponding frequencies 1580 cm^−1^ and 1502 cm^−1^ are taken as the basic reference points [20], and the intensity of different absorption peaks can be determined accordingly. This can be used to determine the intensity of different absorption peaks and thus compare them with each other. The trans stretching vibration of the ether bond (C–O–C) appears at 1040 cm^−1^ and the vibration peak of the epoxy group at 826 cm^−1^.

**Figure 8:**
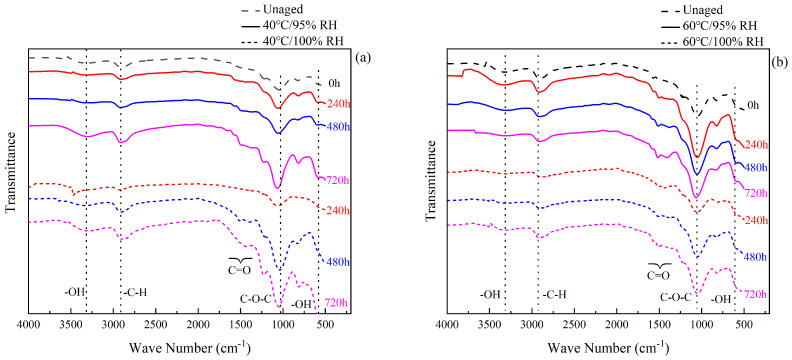
FTIR profiles of EXP2015 after aging: (**a**) 40 °C; (**b**) 60 °C.

The comparative analysis of the FTIR of EXP2015 after aging is shown in Figure 8, which shows that no absorption peaks are shifted, but there is a change in the intensity of absorption peaks. With the increase in hygrothermal aging time, the intensity of the absorption peak at –OH (3320 cm^−1^) significantly increased, which indicated that the absorption of water molecules by the adhesive was caused by hygrothermal aging. The intensity of the –OH absorption peak at 100% RH is greater than that at 95% RH%, both at 40 °C and 60 °C, a finding that validates the accuracy of the moisture absorption process in Section 3.1. The absorption peaks corresponding to the carbonyl group (1604 cm^−1^) and alkyl group (3100~2800 cm^−1^) also increased. A study [35] found that the increase in carbonyl and alkyl groups was due to the hydrolysis of the ester group (R’COOR) under the long-term action of humidity and heat, which indicated that the epoxy resin adhesive might undergo hydrolysis reaction and the transformation of functional groups under the action of humidity and heat aging. The hydrolysis reaction formula is as follows:

(5) $$R^{\prime }COOR+H_{2}O↔R^{\prime }COOH+ROH$$

The process in which water molecules enter the epoxy resin and react with the corresponding hydrophilic groups is known as hydrolysis, which not only destroys the molecular chain of the polymer in the epoxy resin adhesive but also disrupts the cross-linking of ester groups, leading to the generation of new carbonyls or alkyls [35]. Prolonged moisture and heat attack can lead to changes in the chemical composition of the adhesive, causing irreversible damage to the material [45], and according to the hygroscopic analysis in Section 3.1, it can be seen that this process occurs mainly in the first diffusion mechanism. In addition, it was found that the higher the temperature at 100% humidity, the lower the intensity of the vibrational peaks of carbonyl and alkyl groups, suggesting that the post-curing reaction hinders the hydrolysis reaction, which is manifested as a lesser degree of loss in terms of failure strength. In the FTIR spectra, the most obvious fluctuation is the ether bond (C–O–C) trans stretching vibration at 1040 cm^−1^, which is related to the etherification reaction. The hydrogen atom of the hydroxyl group in the alcohol or phenol molecule is replaced by an alkyl group or an aryl group to generate the alcohol ether or the phenol ether, and the higher temperature promotes the occurrence of the etherification reaction. Combined with the analysis in Section 3.2, it can be seen that the generation of etheric substances will cause a decrease in the mechanical properties of the adhesive.

### 3.4. Fracture Analysis of Bonded Areas

#### 3.4.1. Macroscopic Failure Section

**Figure 9:**
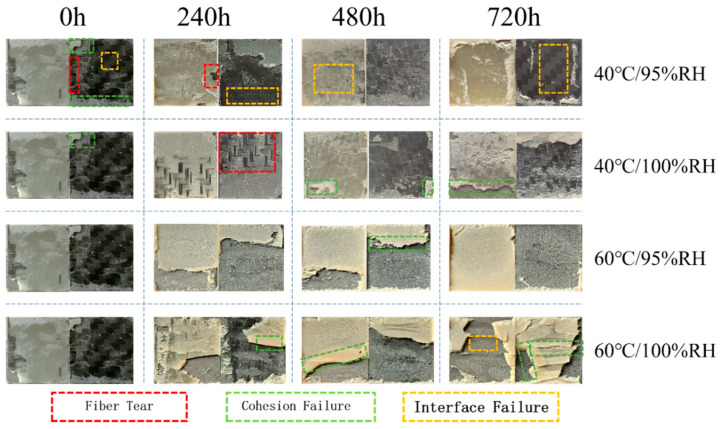
Failure sections of joints in different environments.

Figure 9 shows the macroscopic failure sections of joints during aging in different environments, where the region within the red wireframe is the joint tearing failure region, the green wireframe shows cohesive failure, and the orange wireframe represents interfacial failure. Fiber tearing means that the joint is not damaged, but the damage concerns the bonded material; cohesive failure means that the adhesive joint is damaged from the colloid itself, and there is adhesive residue on the bonding surface; interfacial failure means that the adhesive joint is separated from the bonding surface, and there is no adhesive residue on the interface.

All three forms of failure were found in the bonding area of the unaged joints, with cohesive failure having the largest percentage of the area and interfacial failure the smallest. Under an environment of 40 °C/95% RH, with an increase in aging time, cohesive failure in the adhesive area gradually decreases, and completely disappears at 240 h; on the contrary, the interfacial failure area gradually increases. It has been shown that cohesive failure tends to give full play to the strength of the adhesive layer, while interfacial failure does not play a role in fully exploiting the strength of the adhesive layer, which makes the joints show a gradual decrease in failure strength at 40 °C/95% RH. In addition, under the influence of moisture and heat aging, a thin layer of adhesive appears at the edge of the CFRP substrate, since water molecules enter the bonding area from the bonding line, and higher concentrations of water molecules at the edge weaken the bonding force between polymer molecular chains, which leads to the failure of the adhesive in a part of the area at the edge. A large number of fiber tears were observed in the bonding region aged at 40 °C/100% RH for 240 h. When the applied load was transferred to the continuous fiber layer, the joint exhibited a large value of energy absorption during failure due to the excellent fracture toughness of the continuous carbon fibers. As aging time increases, the failure forms of the joint are still dominated by interfacial failure, and cohesive failure occurs in fewer regions compared to 40 °C/95% RH, showing higher failure intensity.

When the aging temperature was raised to 60 °C, as a result of the temperature effect of the impact, the failure mode of the joints at 60 °C/95% RH was mainly an interfacial failure, with less cohesive failure occurring in the bonding region before 480 h aging time. The joints were aged up to 720 h, at which time the failure mode in the bonding region was transformed into complete interfacial failure, indicating that the adhesion between the adhesive and the CFRP substrate was weakened with the increase in the duration of the moisture and heat attack. In conjunction with Section 3.2.2, it can be seen that joint interface failure corresponds to a lower value of energy absorption compared to the other two forms of failure. Unlike the above three environments, the main failure mode of joints at 60 °C/100% RH is cohesive failure, so the joints in this environment show the highest values in terms of failure strength. It is observed that the area where cohesive failure occurs in joints at 60 °C/95% RH is closer to the end of the substrate, indicating that the stress is concentrated at the end of the bonding area during the stretching process; when the humidity is raised to 100%, the residual stress is released to some extent, and the cohesive failure is closer to the middle of the bonding area.

It can be seen from the above that lower failure strengths and energy absorption values are often accompanied by interfacial failure; cohesive failure is the most desirable failure mode for joints, and high failure strengths can be demonstrated during the failure process; and tearing failure is associated with high energy absorption values.

#### 3.4.2. Microscopic Failure Section

**Figure 10:**
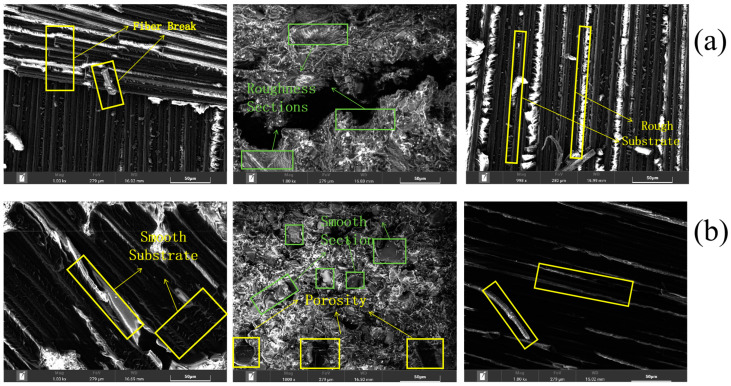
Scanning electron microscopy analysis of tearing and cohesion failure before and after aging: (**a**) no aging; (**b**) aging for 720 h.

To further observe the changes in the bonding region before and after aging, scanning electron microscopy tests were performed on the tearing region and cohesion failure region based on the macroscopic failure section (Figure 10).

**Figure 11:**
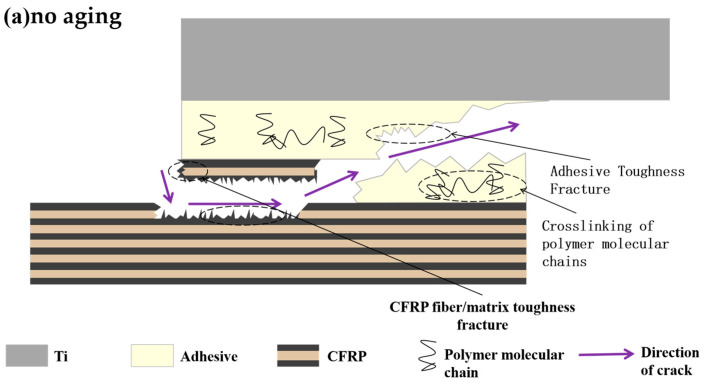

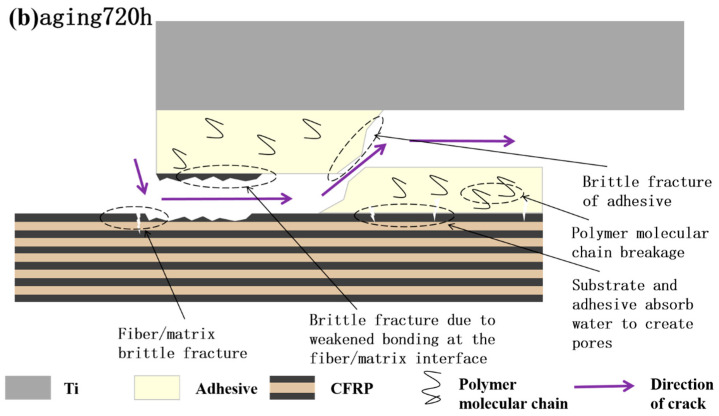
Schematic diagram of joint fracture before and after aging: (**a**) no aging; (**b**) aging for 720 h.

Fiber fracture as well as continuous fiber/matrix debonding can be observed in the tear region before aging. Under high magnification, a large amount of ductile matrix adheres to the surface of continuous fiber, and the surface of continuous fiber/matrix is rough. The failure mode at the tear is mainly the toughness fracture of the fibers and matrix, which suggests that the interface of the continuous fiber/matrix is better bonded with higher fracture energies at the time of failure. The adhesive cohesion failure area shows crack extension, and on both sides of the cracks in the adhesive surface, the fracture is rough, indicating aging. The adhesive internal polymer chain cross-linking density is larger, showing a higher polymerization force and fracture of the energy required to absorb the value. The degree of toughness fracture of the CFRP matrix is reduced after aging, some of the matrix undergoes flatter brittle fracture, and the continuous fiber/matrix debonding interface is smooth (shown in Figure 11b), while traces of fiber breakage are observed in the tear region, indicating that cracks are mainly extend along the continuous fiber/matrix interface. Under actions of humidity and heat, more pores appear on surface of adhesive. This is because water molecules enter the free volume of the adhesive and react with surrounding hydrophilic groups, resulting in the degradation of the adhesive, with the free volume gradually expanding into pores. In addition, some sections of the adhesive are flat and smooth, indicating that the adhesive changes from toughness to plasticity after aging, which is related to the plasticization of the adhesive caused by the humid and hot environment. In conjunction with Section 3.2, it can be seen that when the tear region shows a ductile fracture of the fibers and matrix as well as a ductile fracture of the adhesive by external forces, the failure strength of joints is greater and the energy required for failure is greater. After moisture–heat aging, smooth brittle fracture occurs in the section of the tear region, and the continuous fiber/matrix bonding force appears to be reduced; the plasticizing reaction of the adhesive occurs, and the internal polymerization force of the adhesive appears to be weakened, which leads to a reduction in the failure strength of joints.

Based on the analysis of the microscopic testing of joints before and after aging, the failure mechanism of bonded joints before and after aging is expressed schematically in Figure 11.

During quasi-static tensile failure, cracks in the unaged joints extend between the continuous fiber/matrix layers of the CFRP substrate and within the adhesive, with the main failure modes for the continuous fiber being toughness fracture, continuous fiber/matrix interlayer debonding, adhesive toughness fracture, and the emergence of a small portion of the adhesive failure (interfacial failure). In conjunction with Section 3.3, it can be seen that the polymer molecular chains are more densely cross-linked before aging, which is the reason for the toughness fracture of the adhesive. After aging for 720 h, the joints failed, with cracks expanding along the CFRP matrix as well as within the adhesive, and the main failure modes were a brittle fracture of the CFRP continuous fiber/matrix, brittle debonding between the continuous fiber/matrix, a brittle fracture of the adhesive, and the failure of most of the adhesive. From Section 3.1 and Section 3.2, it can be seen that the process of adhesive water absorption is a chemical mechanism, which results in the breakage of polymer molecular chains, the reduction of cross-linking density, and the brittle fracture of the adhesive. In the failure mode accompanying the crack extension process, the energy required for the ductile fracture of the continuous fibers, CFRP matrix, and adhesive is larger compared to brittle fracture, so the failure strength and energy absorption values of the bonded joints after aging appear to be reduced compared to those without aging. In addition, the adhesive and the substrate absorb water to produce pores, leading to a decline in interfacial adhesion, and the large-area interfacial failure causes the mechanical properties of joints after aging to decline.

### 3.5. Reliability Analysis

Reliability is generally used in the statistical analysis process for the fatigue testing of composite joints [46], and in practical engineering applications, SLJs are often subjected to pure shear stresses which make the product less reliable. A Weibull distribution is used to describe the statistical law of the maximum shear stress value. The probability density function (reliability) of the Weibull distribution is as follows:

(6) $$R\left(\tau \right)=\exp\left[-\frac{{\left(\tau -r\right)}^{m}}{t_{0}}\right]$$

where τ is a stochastic parameter, which, in this section, refers to the maximum shear stress at which the joint fails; *m* is a shape parameter; *r* is a position parameter; and $t_{0}$ is a scale parameter.

In practical engineering applications, the value of the position parameter *r* is often taken to be 0. Therefore, Equation (6) is simplified to a two-parameter distribution, i.e.,

(7) $$R\left(\tau \right)=\exp\left[-\frac{\tau^{m}}{t_{0}}\right]$$

The shape and scale parameters were solved using actual experimental values, and Equation (7) was plotted against the curve.

#### 3.5.1. Reliability Analysis of Joints under 40 °C Ambient Aging

**Figure 12:**
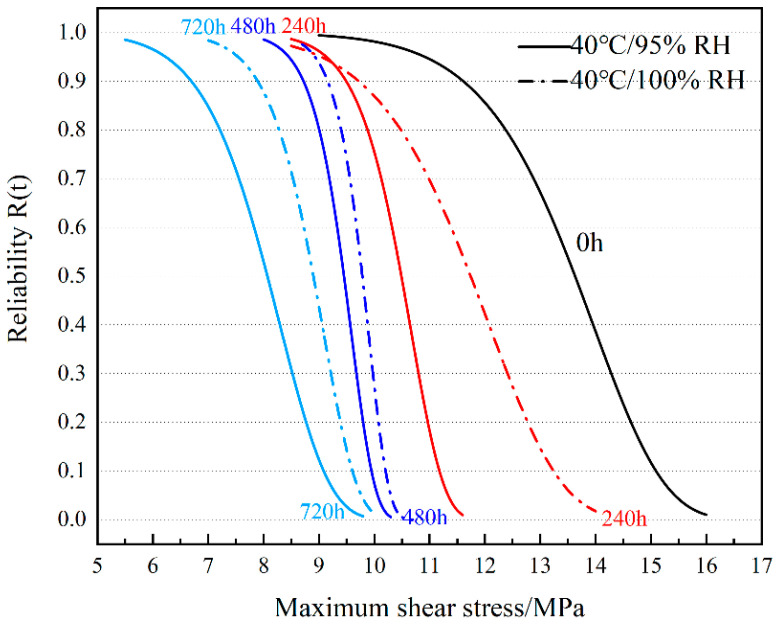
Quasi-static tensile reliability curve of joints at 40 °C aging temperature.

Figure 12 shows the reliability curves of the joints after different aging durations at 40 °C/95% RH and 40 °C/100% RH, where the degradation suffered by the bonded joints during aging is visible. The 0 h curve (unaged joint) shows that the reliability is still greater than 90% when the maximum shear stress $\tau_{max}$ is close to 11.5 MPa. With the gradual increase in $\tau_{max}$ applied to the bonded joint, the reliability of the unaged joint gradually decreases, and the product reaches the median life (50% reliability) when $\tau_{max}$ increases to 13.6 MPa. Under temperature and humidity exposure, the curve shifts towards the region of lower values of $\tau_{max}$, which is related to the degradation of the mechanical properties of the joint. Under 95% RH aging, the 240 h reliability curve shows that $\tau_{max}$ reaches almost 9.5 MPa, and the reliability of the joint is 90%. When the aging time comes to 480 h, the reliability curve appears to shift to the left; at this time, the joint would ideally obtain more than 90% reliability, but in the actual engineering application, the $\tau_{max}$ suffered by the joint shall not exceed 8.7 MPa. The aging 720 h curve shows the most pronounced degradation, with 90% reliability obtained for the joint at a $\tau_{max}$ of 6.7 MPa. The rightward shift of the reliability curve after aging at 100% RH compared to the reliability curve at 95% RH at the same temperature is related to the fact that high humidity can improve the mechanical properties of joints to some extent, as mentioned in Section 3.2 of this paper. Under 100% RH, if the joint is required to have more than 90% reliability under different aging times, its $\tau_{max}$ shall not be lower than 9.7 MPa (240 h), 9.1 MPa (480 h), or 7.9 MPa (720 h) during the application process. It is noteworthy that the curves for different humidity levels at an aging time of 240 h show intersections in the region of high reliability, and the lower the humidity under the same $\tau_{max}$ before the intersection, the higher the reliability; but with the increase in $\tau_{max}$, the advantage of high humidity in terms of reliability appears. From the values and images, it can be seen that the $\tau_{max}$ suffered by joints at 100% humidity is higher than at 95% humidity when the joints obtain rated life (90% reliability) at different aging times. With the increase in $\tau_{max}$ during application, the reliability curves also show the above pattern when the joints reach median life.

#### 3.5.2. Reliability Analysis of Joints under 60 °C Ambient Aging

**Figure 13:**
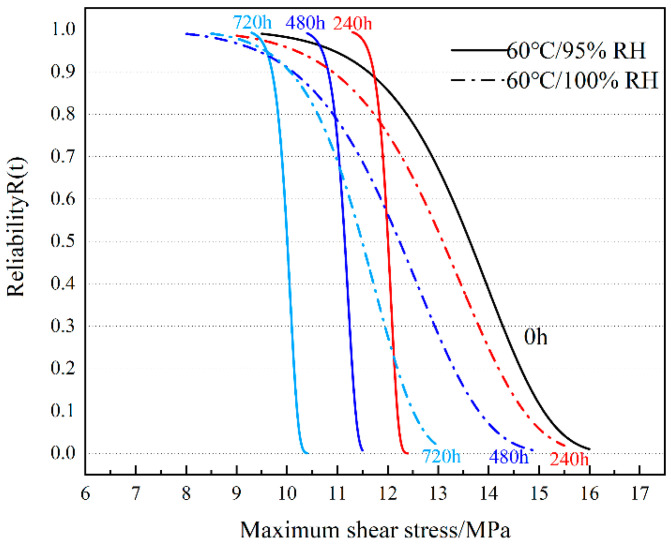
Quasi-static tensile reliability curve of joints at 60 °C aging temperature.

Figure 13 shows the reliability curve of bonded joints in a 60 °C environment. The $\tau_{max}$ suffered by a joint with an aging time of 240 h to obtain 90% reliability at 95% RH is 11.7 MPa, which is comparable to the $\tau_{max}$ suffered by a joint at 0 h to obtain 90% reliability. The $\tau_{max}$ suffered by the joints appeared to increase with aging time, with values of 10.8 MPa (480 h) and 9.7 MPa (720 h), respectively. Compared with the reliability curves at 0 h, the reliability curves in a 95% RH environment are shifted to the low-stress region as a whole, but in the higher reliability region, the aging curves are close to or even appear on the right side of the 0 h curves. The $\tau_{max}$ suffered when the joints obtained 90% reliability in a 100% RH environment were 10.8 MPa (240 h), 10.1 MPa (480 h), and 10.05 MPa (720 h), respectively. According to the images, it can be seen that the $\tau_{max}$ of joints with an aging time of 480 h and 720 h to obtain 90% reliability is not much different, but as $\tau_{max}$ increases, the advantage of joints with a shorter aging time in terms of reliability is highlighted. The slope of the reliability curve is larger at 95% RH, which indicates that the data are less discrete [46]. The slope of the reliability curve is smaller at 100% RH, and the data are more discrete, which indicates that the joints can fail in the low $\tau_{max}$ zone in this environment. However, in the high $\tau_{max}$ region, high humidity corresponds to a larger $\tau_{max}$ for the same reliability.

## 4. Conclusions

In this paper, the failure performance of CFRP-TI bonded joints is investigated by aging them in 40 °C/95% RH, 40 °C/100% RH, 60 °C/95% RH, and 60 °C/100% RH environments for 240 h, 480 h, and 720 h. The following conclusions were drawn:

(1) The adhesive water absorption process is divided into two stages. The first stage is affected by hydrogen bonding, where water molecules diffuse by interacting with hydrophilic functional groups in the epoxy resin; the second stage is related to the aggregation of water molecules, which fill the free volume of the epoxy resin.
(2) From the mechanical property analysis of the joints, it can be seen that the failure strength and energy absorption of the joints at 40 °C/95% RH, 40 °C/100% RH, and 60 °C/95% RH all show different degrees of decrease, while at 60 °C/100% RH, they all show an increase and then a decrease, indicating that there are two factors of post-curing and environmental erosion competing with each other, and the two occur at the same time. When post-curing competes with environmental erosion, the failure load increases; conversely, the failure load decreases; consistent with the results obtained from the FTIR test.
(3) The decline in adhesive performance is due to hydrolysis under the action of moisture and heat aging, destroying the polymer molecular chain stress. The adhesive will also undergo a series of etherification and oxidation reactions. If the temperature is high enough, it will promote the etherification reaction. With the intrusion of moisture and heat, the section toughness fracture gradually shifts to brittle fracture, and the joint failure mode gradually changes from cohesive failure to interfacial failure, resulting in the performance of the adhesive declining.
(4) Compared with before aging, the reliability curve after humidity–heat aging shifts to the low-stress region as a whole, and high humidity can improve the mechanical properties of the joint to some extent.

In summary, the study of the effect of temperature and humidity coupling on the aging failure of carbon fiber composite/titanium alloy bonded joints can promote the innovation and development of bonding technology, which can help to develop more advanced and high-performance bonding materials and technologies, and promote the development of the field of vehicle engineering. This paper only selects a specific humidity–heat coupling environment and an adhesive to study the aging failure mechanism of the joint. The actual life of the vehicle is subject to environmental factors which are complex and variable. In the future, we will study the existing humidity–heat aging mechanism on the basis of more environments and different adhesives, as well as the adhesion mechanism between the substrates.

## Figures and Tables

**Table 1:** CFRP material property parameters [29] (The X direction is the 1 direction, the direction perpendicular to X within the pavement plane is the 2 direction, and the direction perpendicular to the 12 plane is the 3 (normal) direction. E_1_, E_2_, and E_3_ are the moduli of elasticity in the 1, 2, and 3 directions, respectively; V_12_, V_13_, and V_23_ are the Poisson’s ratios of the 12, 13, and 23 surfaces, respectively; G_12_, G_13_, and G_23_ are the shear moduli in the 12, 13, and 23 surfaces, respectively).

$E_{1}\;[GPa]$	$E_{2},E_{3}\;[GPa]$	$v_{12},v_{13}$	$v_{23}$	$G_{12},G_{13}\;[GPa]$	$G_{23}\;[GPa]$
114	8.16	0.3	0.45	4.16	3.0

**Table 2:** Parameters of Araldite^®^2015 [14].

	Araldite^®^2015
Young’s modulus, E [GPa]	1.85
Shear modulus, G [GPa]	0.56
Densities [kg·m^3^]	1.4 × 10^−6^
Poisson’s ratio, V	0.33

**Table 3:** SDF model parameters for EXP2015 in different environments.

Temp (°C)	RH (%)	*D*_1_ (10^−3^ mm^2^/h)	*D*_2_ (10^−3^ mm^2^/h)	*M*_1∞_ (%)	*M*_2∞_ (%)	*M*_∞_ (%)	T d/h
40	95	2.20	0.89	1.92	0.84	2.76	264
	100	2.93	0.72	2.77	1.35	4.12	240
60	95	2.60	0.65	2.21	1.04	3.25	312
	100	6.60	0.92	3.91	2.86	6.77	144

**Table 4:** Main band assignments in FTIR of EXP2015 [20,35].

Wave Number (cm^−1^)	Functional Group
3320	–OH, –NH telescoping vibration
3100~2800	Haloalkyl (–CH_3_, –CH_2_) telescoping vibration
1604	Carbonyl (C=O)
1581, 1502	Quadrant stretching of benzene ring
1434	C–H bending of aliphatic moieties
1242	Aromatic ether telescopic vibration
1040	Ether bond C–O–C trans stretching vibrations
826	Epoxy functional groups

## Data Availability

The data is not available to the public due to restrictions such as privacy or ethics.
